# Outcomes After Sentinel Lymph Node Biopsy and Radiotherapy in Older Women With Early-Stage, Estrogen Receptor–Positive Breast Cancer

**DOI:** 10.1001/jamanetworkopen.2021.6322

**Published:** 2021-04-15

**Authors:** Neil Carleton, Jian Zou, Yusi Fang, Stephen E. Koscumb, Osama Shiraz Shah, Fangyuan Chen, Sushil Beriwal, Emilia J. Diego, Adam M. Brufsky, Steffi Oesterreich, Steven D. Shapiro, Robert Ferris, Leisha A. Emens, George Tseng, Oscar C. Marroquin, Adrian V. Lee, Priscilla F. McAuliffe

**Affiliations:** 1 Women’s Cancer Research Center, UPMC Hillman Cancer Center, Pittsburgh, Pennsylvania; 2 Magee-Womens Research Institute, Pittsburgh, Pennsylvania; 3 Medical Scientist Training Program, University of Pittsburgh School of Medicine, Pittsburgh, Pennsylvania; 4 Department of Biostatistics, University of Pittsburgh, Pittsburgh, Pennsylvania; 5 Clinical Analytics, UPMC Health Services Division, Pittsburgh, Pennsylvania; 6 School of Medicine, Tsinghua University, Beijing, China; 7 Department of Radiation Oncology, UPMC Hillman Cancer Center, Pittsburgh, Pennsylvania; 8 Division of Surgical Oncology, Department of Surgery, University of Pittsburgh School of Medicine, Pittsburgh, Pennsylvania; 9 Division of Medical Oncology, Department of Medicine, University of Pittsburgh School of Medicine, Pittsburgh, Pennsylvania; 10 Department of Pharmacology and Chemical Biology, University of Pittsburgh School of Medicine, Pittsburgh, Pennsylvania; 11 Division of Pulmonary, Allergy, and Critical Care Medicine, University of Pittsburgh School of Medicine, Pittsburgh, Pennsylvania

## Abstract

**Importance:**

Overtreatment of early-stage breast cancer with favorable tumor biology in older patients may be harmful without affecting recurrence and survival. Guidelines that recommend deimplementation of sentinel lymph node biopsy (SLNB) (Choosing Wisely) and radiotherapy (RT) (National Comprehensive Cancer Network) have been published.

**Objective:**

To describe the use rates and association with disease recurrence of SLNB and RT in older women with breast cancer.

**Design, Setting, and Participants:**

This cohort study obtained patient and clinical data from an integrated cancer registry and electronic health record of a single health care system in Pennsylvania. The cohort was composed of consecutive female patients 70 years or older who were diagnosed with early-stage, estrogen receptor–positive, *ERBB2* (formerly *HER2*)–negative, clinically node-negative breast cancer from January 1, 2010, to December 31, 2018, who were treated at 15 community and academic hospitals within the health system.

**Exposures:**

Sentinel lymph node biopsy and adjuvant RT.

**Main Outcomes and Measures:**

Primary outcomes were 5-year locoregional recurrence-free survival (LRFS) rate and disease-free survival (DFS) rate after SLNB and after RT. Secondary outcomes included recurrence rate, subgroups that may benefit from SLNB or RT, and use rate of SLNB and RT over time. Propensity scores were used to create 2 cohorts to separately evaluate the association of SLNB and RT with recurrence outcomes. Cox proportional hazards regression model was used to estimate hazard ratios (HRs).

**Results:**

From 2010 to 2018, a total of 3361 women 70 years or older (median [interquartile range {IQR}] age, 77.0 [73.0-82.0] years) with estrogen receptor–positive, *ERBB2*-negative, clinically node-negative breast cancer were included in the study. Of these women, 2195 (65.3%) received SLNB and 1828 (54.4%) received adjuvant RT. Rates of SLNB steadily increased (1.0% per year), a trend that persisted after the 2016 adoption of the Choosing Wisely guideline. Rates of RT decreased slightly (3.4% per year). To examine patient outcomes and maximize follow-up time, the analysis was limited to cases from 2010 to 2014, identifying 2109 patients with a median (IQR) follow-up time of 4.1 (2.5-5.7) years. In the propensity score–matched cohorts, no association was found between SLNB and either LRFS (HR, 1.26; 95% CI, 0.37-4.30; *P* = .71) or DFS (HR, 1.92; 95% CI, 0.86-4.32; *P* = .11). In addition, RT was not associated with LRFS (HR, 0.33; 95% CI, 0.09-1.24; *P* = .10) or DFS (HR, 0.99; 95% CI, 0.46-2.10; *P* = .97). Subgroup analysis showed that stratification by tumor grade or comorbidity was not associated with LRFS or DFS. Low absolute rates of recurrence were observed when comparing the groups that received SLNB (3.5%) and those that did not (4.5%) as well as the groups that received RT (2.7%) and those that did not (5.5%).

**Conclusions and Relevance:**

This study found that receipt of SLNB or RT was not associated with improved LRFS or DFS in older patients with ER-positive, clinically node-negative breast cancer. Despite limited follow-up time and wide 95% CIs, this study supports the continued deimplementation of both SLNB and RT in accordance with the Choosing Wisely and National Comprehensive Cancer Network guidelines.

## Key Points

**Question:**

What is the rate of sentinel lymph node biopsy (SLNB) and adjuvant radiotherapy (RT) use in women 70 years or older with early-stage, estrogen receptor–positive, *ERBB2* (formerly *HER2*)–negative, clinically node-negative breast cancer, and what are the recurrence outcomes?

**Findings:**

In this cohort study of 2109 older women with early-stage, estrogen receptor–positive breast cancer, rates of SLNB and RT use remained high and were increasing for SLNB. Treating physicians tended to select healthier patients with fewer comorbidities for SLNB and RT, but receipt of either SLNB or RT was not associated with improvements in locoregional recurrence-free or disease-free survival.

**Meaning:**

Results of this study suggest that deimplementation of both SLNB and RT interventions should be strongly considered in older patients with estrogen receptor–positive, clinically node-negative breast cancer.

## Teaser

This cohort study evaluates the continued use of sentinel lymph node biopsy for axillary staging in older patients with early-stage breast cancer and greater likelihood of having more comorbidities and functional limitations.

## Introduction

As the population in the United States ages, the incidence of cancers in older patients is estimated to increase, and these cancers are expected to account for nearly 70% of all cases diagnosed by 2030.^1^ A critical risk factor for the development of breast cancer is age, with most of those diagnosed being older than 65 years and nearly 20% being older than 75 years.^1^ Few guidelines exist for the clinical treatment of older patients with breast cancer because of their lack of representation in randomized clinical trials. Most breast cancer randomized clinical trials exclude women who are older, have existing comorbidities, and/or are cognitively impaired.^2,3^ Oncologists often make treatment decisions with uncertainty in this age group.

Managing breast cancer in older adults brings challenges for physicians. Although many treatment-associated morbidities may be low in younger women with breast cancer, older patients are more prone to adverse effects that can have a substantial impact on quality of life and the ability to perform functions of daily living.^4^ Older populations can be subjected to both overtreatment and undertreatment^5^; because they are more likely to have comorbidities and functional limitations, physicians are less likely to treat cancers aggressively, leading to increased risk for locoregional recurrence and decreased overall survival.^4,6,7^ Conversely, overtreatment of a cancer that ultimately will not lead to death subjects these patients to unnecessary tests and procedures.^8,9^

To avoid overtreatment, the Society of Surgical Oncology (SSO) adopted the Choosing Wisely guideline established by the American Board of Internal Medicine Foundation, which recommends against the routine use of sentinel lymph node biopsy (SLNB) for axillary staging in patients older than 70 years with early-stage, hormone receptor–positive, clinically node-negative breast cancer.^10^ However, adherence to this guideline remains low, given that, in this population of patients, nearly 60% to 80% receive SLNB and there is conflicting evidence on the patterns and perceived benefits of SLNB use.^9,11,12,13^ Despite the Choosing Wisely guideline, limited conclusive evidence exists for definitive omission because no trials have performed a head-to-head comparison of SLNB to no axillary surgery in older patients.^14,15,16,17,18,19,20^ A review on deimplementation of SLNB in breast cancer among older adults noted the analysis of SLNB in 2 small institutional studies (sample of 124 patients for 1 study^15^ and 178 patients for the other^16^) and the National Cancer Database (including thousands of aggregated patients), but large institutional data are lacking.

In addition, the National Comprehensive Cancer Network (NCCN) guideline indicates that deimplementation of radiotherapy (RT) in this group of patients may be feasible.^21^ Strong evidence generated by the Cancer and Leukemia Group B (CALGB) 9343 trial^22^ with long-term follow-up^23^ show that adjuvant therapy with tamoxifen citrate alone without the addition of RT is a feasible treatment strategy for older patients with early-stage, estrogen receptor (ER)–positive breast cancer. Despite this evidence, there has been limited change in clinical practice and rates of RT use continue to be high.^21,24^

This study aimed to retrospectively describe the use rates and association with disease recurrence of SLNB and RT in women aged 70 years or older with early-stage, ER-positive, *ERBB2* (formerly *HER2*)–negative, clinically node-negative breast cancer. Unlike previous studies on this topic, this study used highly annotated data derived from the cancer registry and electronic health record of a multisystem academic and community health care network to evaluate the feasibility of further deimplementation of SLNB and RT in this patient population.

## Methods

### Data Source

This retrospective cohort study included female adults (aged ≥18 years) who were diagnosed with breast cancer from January 1, 2010, to December 31, 2018, and treated at 15 community and academic hospitals within a single health care system in Pennsylvania. The University of Pittsburgh Institutional Review Board approved this study and waived informed consent because all of the data used were deidentified. We followed the Strengthening the Reporting of Observational Studies in Epidemiology (STROBE) reporting guideline.

Clinical data used in this study were obtained from the UPMC Network Cancer Registry. In general, these data are retrospectively manually abstracted according to the data standards of the North American Association of Central Cancer Registries and include data of patients seen across the health care system. Collected clinical data included age at diagnosis, clinical and pathological TNM staging, axillary staging procedure, breast surgical procedure, and adjuvant therapy (RT, hormone therapy, or chemotherapy). Comorbidity data were derived from appropriate *International Classification of Diseases, Ninth Revision, Clinical Modification,* and *International Statistical Classification of Diseases, Tenth Revision, Clinical Modification,* diagnosis codes. The specific codes that were used to derive patient-specific comorbidities can be found in eTable 1 in the Supplement.

### Study Population, Outcomes, and Variable Definitions

We identified women with ER-positive, *ERBB2*-negative invasive breast cancer in the UPMC network between January 1, 2010, and December 31, 2018 (n = 7328). From this cohort, we selected those who were 70 years or older and diagnosed with ER-positive, clinically node-negative breast cancer between January 1, 2010, and December 31, 2018, to examine rates of SLNB and RT. Furthermore, to allow for adequate follow-up time, we limited the analysis to cases diagnosed between January 1, 2010, and December 31, 2014 (n = 2109), to explore patient outcomes.

The primary outcomes in this study were 5-year locoregional recurrence-free survival (LRFS) rate and disease-free survival (DFS) rate after SLNB as well as LRFS and DFS after RT. Secondary outcomes included the factors associated with recurrence, potential subgroups that may benefit from SLNB or RT, and the trends in SLNB and RT use in this patient population over time.

Locoregional recurrence-free survival was defined as the time from diagnosis to the time to an event, including a local or regional recurrence or censoring if lost to follow-up. Disease-free survival was defined as the time from diagnosis to the time of any disease recurrence; neither LRFS nor DFS include second primary cancers. Non-breast cancer–specific mortality events that occurred before a recurrence were considered censored events. Modified Charlson Comorbidity Index (mCCI score) was calculated with a weighted sum of comorbidities (same weights that are used to calculate the original Charlson Comorbidity Index score)^25,26^; eTable 2 in the Supplement shows the weights for each comorbidity captured in this data set.

### Statistical Analysis

To minimize the potential bias of treatment allocation and confounding on the estimation of the association of treatment with LRFS and DFS, we conducted a propensity score–matching analysis. The clinical differences between SLNB and RT necessitate different analyses and outcomes; thus, we matched patients according to different variables for SLNB and RT, creating 2 different cohorts (A and B) to isolate the associations of SLNB and RT (eFigure 1 in the Supplement). In cohort A, in which SLNB outcomes were evaluated, we matched patients on the basis of age, comorbidity score, cancer stage, tumor grade, receipt of mastectomy, receipt of endocrine therapy, receipt of chemotherapy, and site of treatment. In cohort B, in which RT outcomes were evaluated, we matched patients on the basis of the aforementioned variables along with receipt of SLNB and pathological node status, which is information known after a patient undergoes SLNB. Given these variables, we performed propensity score matching with a 5:1 nearest-neighbor matching strategy and a caliper of 0.2 times the SD of the propensity score.

In the propensity score–matched cohorts, we performed subgroup analyses. In both cohorts A and B, patients who did or did not undergo SLNB and RT were further stratified by tumor grade and comorbidity status. Log-rank testing was used to assess LRFS and DFS between these groups.

All continuous data were expressed as median (interquartile range [IQR]) unless otherwise specified. Categorial variables were expressed as frequencies (%). Descriptive data of patient characteristics were compared using either the Mann-Whitney test, χ^2^ test, or Fisher exact test. Sankey diagrams were used to show the proportional relationships between the various interventions that patients could have undergone. Kaplan-Meier survival curves compared DFS and LRFS. Multivariable Cox proportional hazards regression modeling was used to calculate hazard ratios (HRs), which were adjusted for age, tumor grade, cancer stage, comorbidity score, patient income, area deprivation index, and insurance status.

A 2-sided *P* < .05 was considered statistically significant, and CIs were reported for 95th percentiles. Propensity score matching was performed using Stata, V.16 (StataCorp LLC), and all other statistical analyses were conducted in R, version 3.6 (R Foundation for Statistical Computing).

## Results

### Rates of SLNB and RT Use

**Figure 1.**
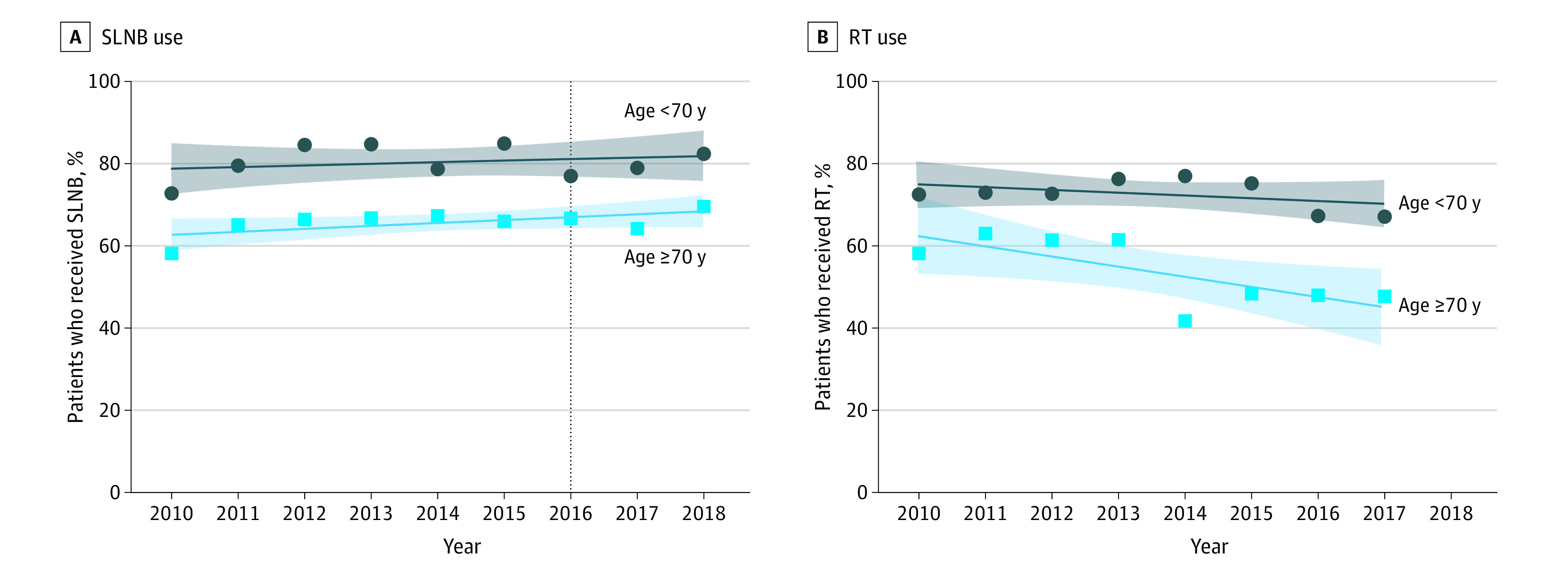
Rates of Sentinel Lymph Node Biopsy (SLNB) and Radiotherapy (RT) Use, 2010-2018 Use rate was calculated as a percentage of patients who underwent SLNB or RT over the total number of patients who did not undergo axillary staging or RT. A, The vertical dashed line at 2016 indicates the adoption by the Society of Surgical Oncology of the Choosing Wisely guideline. The *P* values for patients who received SLNB were *P* = .09 for those younger than 70 years and *P* = .49 for those aged 70 years or older. B, Limited data were available for 2018. The *P* values for patients who received RT were *P* = .27 for those younger than 70 years and *P* = .04 for those aged 70 years or older.

Of the 3361 consecutive women 70 years or older with ER-positive, *ERBB2*-negative, clinically node-negative breast cancer identified during the study period (2010-2018), 2195 (65.3%) received SLNB and 1828 (54.4%) received adjuvant RT. Rates of SLNB steadily increased (1.0% per year), a trend that persisted in 2017 and 2018 even after the 2016 SSO adoption of the Choosing Wisely guideline (Figure 1A). During the same period, rates of RT decreased (3.4% per year) (Figure 1B). As a comparison, we studied rates of both SLNB and RT use in women younger than 70 years with ER-positive, *ERBB2*-negative, clinically node-negative breast cancer who were diagnosed over the same period. We observed the same trends with SLNB (increasing use) and RT (decreasing use) over this period.

### Patient Characteristics

In an analysis limited to cases from 2010 to 2014, we identified 2109 consecutive women aged 70 years or older with ER-positive, *ERBB2*-negative, clinically node-negative breast cancer with a median (IQR) follow-up time of 4.1 (2.5-5.7) years (eTable 3 in the Supplement). The median (IQR) age was 77.0 (73.0-82.0) years.

A total of 1373 patients (65.1%) received SLNB and 1219 patients (57.8%) received adjuvant RT. Patients who did not undergo SLNB or RT compared with those who did were older (median age [IQR] at diagnosis, 80.0 [74.0-86.0] years vs 75.0 [72.0-79.0] years; *P* < .001), had shorter median (IQR) follow-up times (3.4 [1.7-5.1] years vs 4.3 [3.1-6.0] years; *P* < .001), had a larger median (IQR) tumor size (15.0 [9.8-27.0] mm] vs 14.0 [9.0-20.0] mm; *P* < .001), had higher mean (SD) mCCI scores (2.7 [1.1] vs 2.5 [0.8]; *P* < .001), had varied treatment courses, and had differences in treatment sites. Consistent with the selection of early-stage breast cancer with favorable tumor biology, we observed low rates of local (n = 28 [1.2%]) and distant (n = 53 [2.5%]) recurrences in this cohort. Furthermore, low absolute rates of recurrence were observed when comparing the groups that received SLNB (3.5%) and those that did not (4.5%) as well as the groups that received RT (2.7%) and those that did not (5.5%). Only 159 patients (11.5%) who underwent SLNB were deemed as having pathologically positive sentinel lymph nodes.

**Figure 2.**
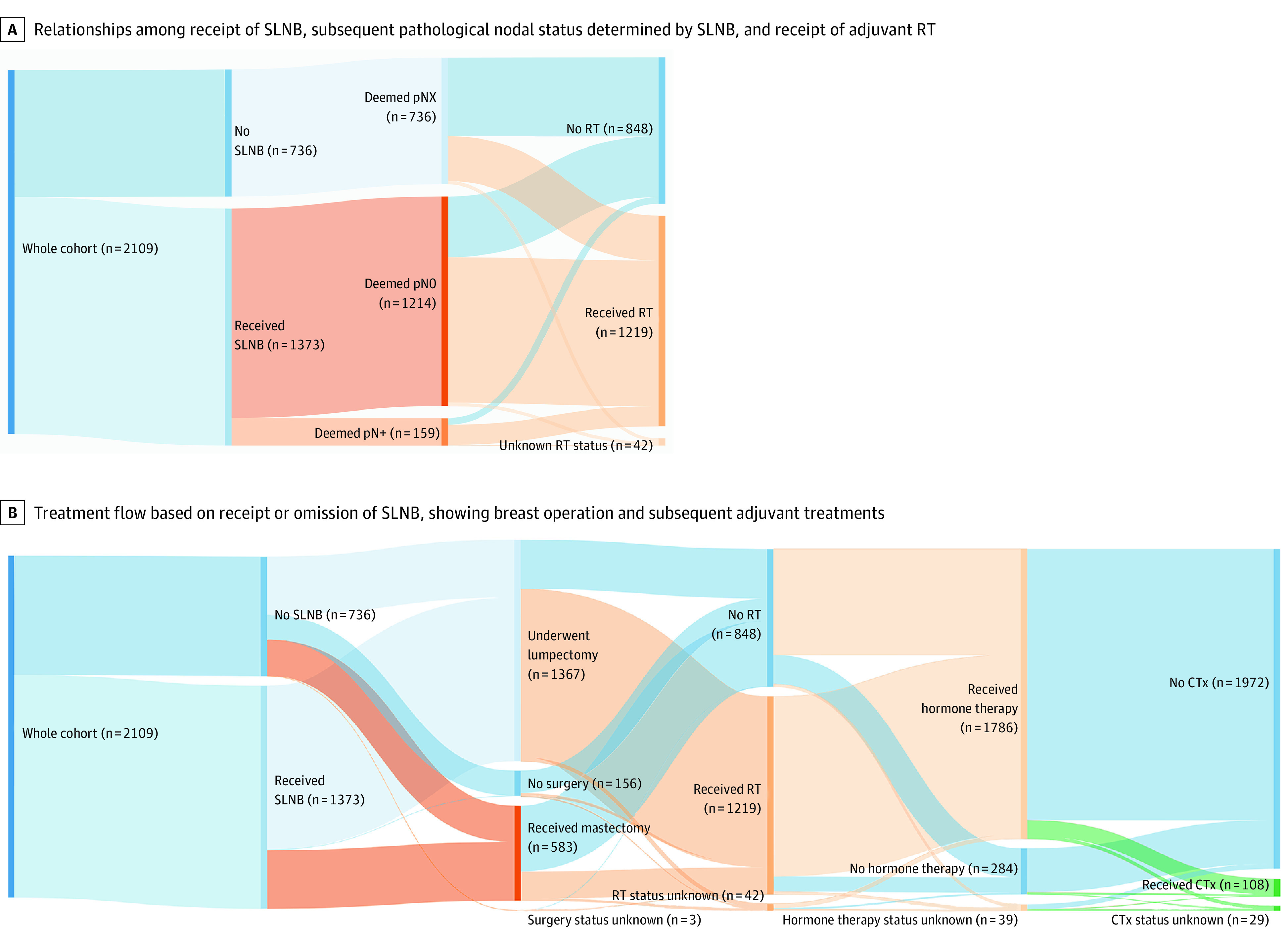
Sankey Diagrams of Treatment and Pathological Relationships in the Study Cohort Panels should be read from left to right. A, Of the whole cohort of 2109 patients, 736 did not undergo sentinel lymph node biopsy (SLNB) but 1373 patients received it. Furthermore, of the 1373 patients who received SLNB, 1214 were deemed pathologically node negative (pN0) and 159 were deemed pathologically node positive (pN+). pNX indicates unknown pathological nodal status in patients who did not receive SLNB. B, Breast operation included lumpectomy, mastectomy, or no surgery, and subsequent adjuvant treatments included radiotherapy (RT), endocrine therapy, and chemotherapy (CTx).

In the propensity score–matched analysis, 965 patients were included in cohort A and 833 patients were included in cohort B. Propensity score matching improved the balance between variables in the matched cohorts compared with the nonmatched cohorts. We prioritized matching for age and mCCI score, which were hypothesized to be the most important factors (standardized difference <0.1 for both cohorts A and B; Table 1). The clinical pathways in this patient cohort and use of axillary staging, surgery (lumpectomy and mastectomy), and adjuvant therapy (RT, hormone therapy, or chemotherapy) are shown in Figure 2A and B.

**Table 1.** Standardized Differences in Matched and Nonmatched Cohorts

Variable	Cohort A matching to evaluate SLNB		Cohort B matching to evaluate RT	
	Nonmatched group	Matched group	Nonmatched group	Matched group
Age	0.604	0.033	0.609	0.036
mCCI score	0.200	0.071	0.083	0.083
Cancer stage	0.322	0.041	0.248	0.190
Tumor grade	0.267	0.222	0.093	0.215
Pathological node status^a^	NA	NA	0.150	0.061
Receipt of				
SLNB	NA	NA	0.488	0.107
Hormone therapy	0.115	0.166	0.277	0.011
Any surgery	0.377	0.032	1.184	0.099
Chemotherapy	0.03	0.11	0.15	0.13
Treatment	0.18	0.04	0.57	0.17

Abbreviations: mCCI, modified Charlson Comorbidity Index; NA, not applicable; RT, radiotherapy; SLNB, sentinel lymph node biopsy.

^a^ Pathological node status and receipt of SLNB were used only to match cohort B.

### Association Between Receipt of SLNB or RT and Disease Recurrence

**Figure 3.**
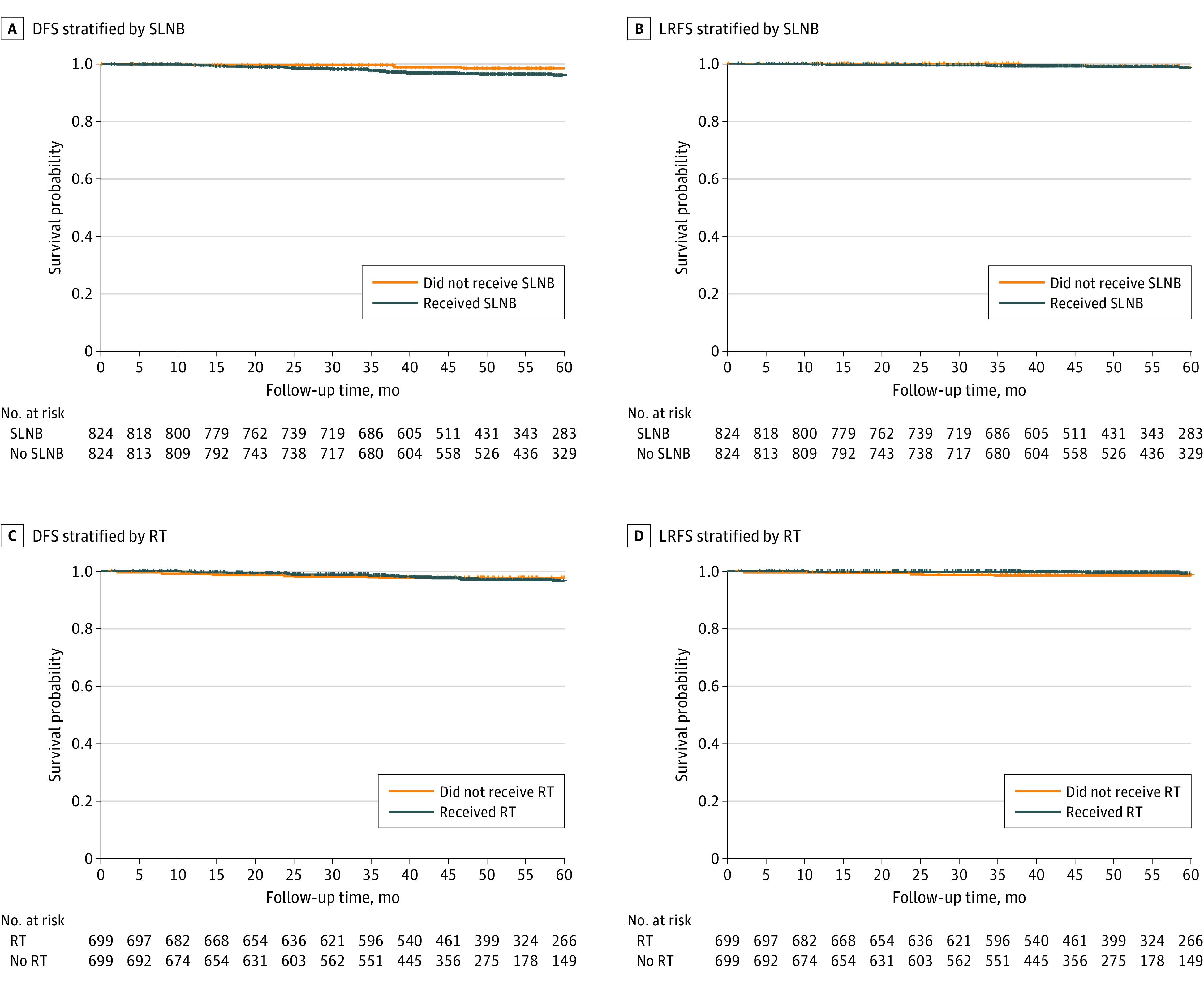
Kaplan-Meier Recurrence-Free Survival Estimates of Patients Who Underwent Sentinel Lymph Node Biopsy (SLNB) and Radiotherapy (RT) in the Propensity Score–Matched Cohorts Patients who did not die and were lost to follow-up within the observation period were censored at their date of last contact. DFS indicates disease free survival; LRFS, locoregional recurrence-free survival.

In cohort A, a non–statistically significant difference in DFS was found in patients who did vs those who did not undergo SLNB, with the patients who did not receive SLNB having an improved DFS (Figure 3A). No overall difference in LRFS was found (Figure 3B). In multivariable Cox proportional hazards regression modeling, SLNB was not associated with DFS (HR, 1.92; 95% CI, 0.86-4.32; *P* = .11) or LRFS (HR, 1.26; 95% CI, 0.37-4.30; *P* = .71) (Table 2).

**Table 2.** Multivariable Analysis for Locoregional Recurrence-Free Survival (LRFS) and Disease-Free Survival (DFS) Using Cox Proportional Hazards Regression Modeling

Variable	LRFS		DFS	
	HR (95% CI)	*P* value	HR (95% CI)	*P* value
**Results for cohort A matching to evaluate SLNB**				
SLNB	1.26 (0.37-4.30)	.71	1.92 (0.86-4.32)	.11
Age	0.99 (0.88-1.11)	.87	1.05 (0.98-1.12)	.14
mCCI score	1.63 (0.98-2.69)	.06	1.34 (0.97-1.85)	.08
Grade 2 vs grade 1 disease	2.08 (0.45-9.68)	.35	2.90 (0.85-9.87)	.09
Grade 3 vs grade 1 disease	3.35 (0.41-27.00)	.26	6.27 (1.68-23.00)	.006
T2 vs T1 tumor	1.58 (0-100)	.89	11.84 (1.57-89.00)	.02
T3 vs T1 tumor	30.31 (0-100)	.29	30.39 (3.47-100)	.002
**Results for cohort B matching to evaluate RT**				
RT	0.33 (0.09-1.24)	.10	0.99 (0.46-2.10)	.97
Pathological node status^a^	NA	NA	0.86 (0.26-2.86)	.81
Age	1.19 (1.05-1.35)	.007	1.20 (1.11-1.30)	<.001
mCCI score	1.26 (0.54-2.92)	.59	1.35 (0.89-2.08)	.16
Grade 2 vs grade 1 disease	0.42 (0.10-1.78)	.24	1.36 (0.48-3.86)	.56
Grade 3 vs grade 1 disease	0.25 (0.03-2.50)	.24	1.55 (0.46-5.18)	.48
T2 vs T1 tumor	1.65 (0-100)	.93	12.15 (1.32-100)	.03
T3 vs T1 tumor	8.47 (0-100)	.69	5.77 (1.15-100)	.04

Abbreviations: HR, hazard ratio; mCCI, modified Charlson Comorbidity Index; NA, not applicable; RT, radiotherapy; SLNB, sentinel lymph node biopsy.

^a^ For the LRFS statistics in the pathological node status variable, there was an inadequate sample size of cases with LRFS and positive pathological node status to derive HR and *P* value.

Radiotherapy included whole breast RT with coverage of level 1 and 2 lymph node regions in tangential beam for patients with a positive SLNB result or patients in whom SLNB was not performed. The most common course of RT was external beam with hypofractionation (40-42.5 Gy in 15-16 fraction, with an option for 10 Gy boost in 4-5 fraction).

In cohort B, no significant difference in DFS was found in patients who did vs did not receive RT (Figure 3C). Similarly, no significant difference in LRFS was found for those who received vs those who did not receive RT (Figure 3D). In the multivariable analysis, RT again did not have a significantly lower hazard for either DFS (HR, 0.99; 95% CI, 0.46-2.10; *P* = .97) or LRFS (HR, 0.33; 95% CI, 0.09-1.24; *P* = .10) (Table 2). In addition, having a positive SLNB result was not associated with DFS.

We also observed that a proportion of women included in this analysis had undergone a mastectomy (36.0% [n = 45] in the group that did not receive RT vs 11.0% [n = 78] in the group that received RT). Thus, to better determine if RT can be omitted after conservative surgery specifically, we performed a similar analysis in this propensity score–matched cohort by excluding the patients who underwent a mastectomy. We found similar results in that RT did not improve LRFS or DFS on both log-rank testing and multivariable analysis (eFigure 2 in the Supplement).

### Subgroup Analysis

We performed a subgroup analysis to ascertain whether a specific group of patients may benefit from either SLNB or RT. For SLNB, when stratifying by both receipt of SLNB and tumor grade (grade 1 and grade ≥2), we found no difference in LRFS on log-rank testing (did not receive SLNB, grade 1: 0 recurrence and grade ≥2: 6 recurrences vs received SLNB, grade 1: 2 recurrences and grade ≥2: 8 recurrences; *P* = .44). When stratifying by both receipt of SLNB and comorbidity score (mCCI score), we found a significant difference in LRFS on log-rank testing (did not receive SLNB, mCCI score of 3: 6 recurrences; received SLNB, mCCI score of 2: 5 recurrences; received SLNB, mCCI score of 3: 5 recurrences; all other group combinations had 0 recurrence; *P* = .001). This difference was driven by patients with a low mCCI score who underwent SLNB and subsequently had locoregional recurrences.

For RT, when stratifying by both receipt of radiation and tumor grade, we found no differences in survival on log-rank testing for either LRFS (did not receive RT, grade 1: 3 recurrences and grade ≥2: 4 recurrences vs received RT, grade 1: 1 recurrence and grade ≥2: 3 recurrences; *P* = .23) or DFS (did not receive RT, grade 1: 3 recurrences and grade ≥2: 4 recurrences vs received RT, grade 1: 2 recurrences and grade ≥2: 12 recurrences; *P* = .20). When stratifying by both receipt of RT and comorbidity score (mCCI score), we found no differences in survival on log-rank testing for either LRFS (did not receive RT, mCCI score of 2: 6 recurrences; did not receive RT, mCCI score of 3: 1 recurrence; received RT, mCCI score of 2: 1 recurrence; received RT, mCCI score of 3: 3 recurrences; all other group combinations had 0 recurrence; *P* = .51) or DFS (did not receive RT, mCCI score of 2: 6 recurrences; did not receive RT, mCCI score of 3: 1 recurrence; received RT, mCCI score of 2: 6 recurrences; received RT, mCCI score of 3: 7 recurrences; received RT, mCCI score of 5: 1 recurrence; all other group combinations had 0 recurrence; *P* = .17). These findings further support the possibility that both SLNB and RT can be omitted for all patients, regardless of tumor grade or comorbidity status.

## Discussion

In this retrospective, propensity score–matched cohort study, rates of SLNB and RT in women 70 years or older with early-stage breast cancer remained high. Multivariable analysis showed no difference in LRFS or DFS for those who did vs did not receive SLNB. Similarly, receipt of RT in this population was not associated with improved LRFS and DFS. These results indicate that both SLNB and RT should continue to be deimplemented in accordance with current guidelines.

In 2016, the SSO formally adopted recommendations from the Choosing Wisely guideline, one of which was to avoid the routine use of SLNB in women 70 years or older with early-stage, hormone receptor–positive, *ERBB2*-negative, clinically node-negative breast cancer. This adoption was based on posthoc analyses from trials conducted in the past decade, although these trials were not randomized to compare SLNB with no axillary evaluation.^27,28,29,30^ Recent studies have described the challenges in deimplementation of SLNB, citing surgeons who described a “lack of familiarity” with or skepticism toward the recommendation as the key reasons for continued high rates of SLNB use.^31^ Other barriers to deimplementation include malpractice concerns, patient demands, and the need for additional evidence before changing practice.^32^ These results suggest that further studies are needed to support deimplementation.

We believe that the present study, which used aggregated data from 15 community and academic hospitals within a single health care system and had more than 10 times the number of cases than in previous studies, provides early evidence that use of SLNB has remained high in breast cancer among older adults after the SSO adoption of the Choosing Wisely guideline in 2016. The results suggested that confounding by indication is a high possibility for healthier patients who undergo the procedure.^33^ Healthier patients tend to be selected for the procedure, do better because of their general state of health, and are followed up more closely and for a longer time. Thus, we tried to control for this confounding by conducting propensity score matching. Propensity score matching resulted in well-matched cohorts, especially with respect to age and comorbidity, which were hypothesized to be the most critical variables to control for confounding. After the matching, we found that receipt of SLNB was not associated with improvements in either LRFS or DFS. In addition, we found that patients who received SLNB had a low rate of pathologically positive sentinel lymph nodes (11.5%), which is consistent with rates reported in previous studies.^11,34^

In the multivariable analysis, we showed that even having a pathologically positive sentinel lymph node was not significantly associated with either DFS or LRFS, indicating that getting this information from SLNB is not warranted. In total, these data strongly support the continued deimplementation of SLNB in this patient population in accordance with the Choosing Wisely guideline recommendation.

The NCCN recommends considering the omission of RT in this patient population if these patients are treated with adjuvant systemic endocrine therapy. The NCCN guidelines were implemented after the landmark CALGB 9343 trial,^22^ which showed that adjuvant tamoxifen alone was a sufficient treatment for older patients with early-stage ER-positive disease, supporting the omission of adjuvant RT. However, despite this trial, long-term follow-up of this trial, and a change in the NCCN guideline to support RT omission, rates of RT have remained high for this cohort of older patients.^24,35,36^ This study captured data on patients at a different period (2010-2018) from the study period (late 1990s to early 2000s) of the CALGB 9343 trial. Thus, advances in endocrine therapies and radiation treatments have occurred since the CALGB 9343 trial. We surmised that these advances could be a reason that rates of RT remain high despite previous evidence.

Although the results of this study were not necessarily different from those of the CALGB 9343 trial, it indicated that more data are needed to support the continued deimplementation of RT. Similar to SLNB, RT was not statistically significantly associated with DFS or LRFS on multivariable analysis. In addition, similar to the CALGB 9343 trial^22,23^ and other studies,^37,38^ this study also found low absolute rates of both locoregional and distant recurrences in the groups that underwent RT vs the groups that did not undergo RT. Thus, given the propensity score–matched data, we found that omission of RT remains a safe option in older patients with ER-positive disease.

### Limitations

This study has limitations. First, the data did not capture predicted life expectancy, Eastern Cooperative Oncology Group performance status, socioeconomic status, or performance status, which are not readily found within our health system’s electronic health record. Given that these factors were not considered in the propensity score matching or multivariable analyses, there is the potential for residual confounding outside of the mCCI score used in this study to associate comorbidities with recurrence. Furthermore, misclassification or underreporting of data could have occurred if patients sought health care outside of the system, which would not have been captured in our data.^39^ Second, the follow-up time for the patients in the present study was limited; long-term follow-up may be required to assess for late recurrences that often occur with ER-positive tumors. However, the long-term follow-up from the CALGB 9343 trial^21^ showed that, although new recurrences were detected in their study cohorts, these recurrences did not change the conclusion of their original trial results.^16^

## Conclusions

Despite limited follow-up time and wide CIs in the survival analysis, the results of this study suggest the following: (1) SLNB can safely be omitted, in accordance with the Choosing Wisely guideline recommendation, in older patients with ER-positive, clinically node-negative breast cancer; (2) RT can safely be omitted, in accordance with results of the CALBG 9343 trial and NCCN guideline, in older patients with ER-positive, clinically node-negative breast cancer on the basis of LRFS and DFS; (3) rates of RT and SLNB remain high, suggesting that additional studies are needed to investigate why this is the case; and (4) in accordance with the CALGB 9343 trial, low rates of locoregional recurrence with or without RT and low rates of pathological node positivity after SLNB were found, adding further support for the omission of both interventions.

## Notes

Supplement. **eTable 1.** ICD-9 and ICD-10 Codes Used to Define Comorbidities **eTable 2.** Calculation of Modified Charlson Comorbidity Index **eTable 3.** Baseline Characteristics of Patients **eFigure 1.** Flowchart for the Derivation of the Propensity Score Matched Cohorts **eFigure 2.** Re-analysis of Cohort B Excluding Those Patients That Underwent Mastectomy
