# Supplementary material for: Outcomes After Sentinel Lymph Node Biopsy and Radiotherapy in Older Women With Early-Stage, Estrogen Receptor–Positive Breast Cancer
Source: JAMA Netw Open. 2021 Apr 15;4(4):e216322. doi: 10.1001/jamanetworkopen.2021.6322 (PMC8050744; doi:10.1001/jamanetworkopen.2021.6322)
Supplement: Supplement. — eTable 1. ICD-9 and ICD-10 Codes Used to Define Comorbidities eTable 2. Calculation of Modified Charlson Comorbidity Index eTable 3. Baseline Characteristics of Patients eFigure 1. Flowchart for the Derivation of the Propensity Score Matched Cohorts eFigure 2. Re-analysis of Cohort B Excluding Those Patients That Underwent Mastectomy [file jamanetwopen-e216322-s001.pdf]

Supplementary Online Content

Carleton N, Zou J, Fang Y, et al. Outcomes after sentinel lymph node biopsy and radiotherapy in older women with early-stage, estrogen receptor–positive breast cancer. *JAMA Netw Open*. 2021;4(4):e216322. doi:10.1001/jamanetworkopen.2021.6322

eTable 1. ICD-9 and ICD-10 Codes Used to Define Comorbidities

eTable 2. Calculation of Modified Charlson Comorbidity Index

eTable 3. Baseline Characteristics of Patients

eFigure 1. Flowchart for the Derivation of the Propensity Score Matched Cohorts

eFigure 2. Re-analysis of Cohort B Excluding Those Patients That Underwent Mastectomy

This supplementary material has been provided by the authors to give readers additional information about their work.

© 2021 Carleton N et al. *JAMA Network Open*

eTable 1. ICD-9 and ICD-10 Codes Used to Define Comorbidities

Comorbidity	ICD-9 Codes	ICD-10 Codes
Congestive heart failure	398.91, 402.01, 402.11, 402.91, 404.01, 404.03, 404.11, 404.13, 404.91, 404.93, 415.0, 416.0-1, 416.8-417.1, 417.8-9, 425.0, 425.11, 425.18, 425.2-9, 428.0-23, 428.30-33, 428.40-43, 428.9-429.1	A18.84, I09.81, I11.0, I13.0, I13.2, I26.01-02, I26.09-127.2, I27.81, I27.89-9, I28.0-1, I28.8-9, I42-3, I50.1, I50.20-23, I50.30-33, I50.40-43, I50.9, I51.4-5
Vascular disease	410.01-02, 410.11-12, 410.21-22, 410.31-32, 410.41-42, 410.51-52, 410.61-62, 410.71-72, 410.81-82, 410.91-92, 412, 440.21, 440.30-32, 440.8-9, 444.01, 444.09, 444.1, 444.21-22, 444.81, 444.89-90, 445.01-02, 445.81, 445.89	I21.01-02, I21.09, I21.11, I21.19, I21.21, I21.29, I21.3-4, I22.0-2, I22.8-9, I25.2, I70.211-213, I70.218-219, I70.301-303, I70.308-309, I70.311-313, I70.318-319, I70.321-323, I70.328-329, I70.331-335, I70.338-339, I70.341-345, I70.348-349, I70.35, I70.361-363, I70.368-369, I70.391-393, I70.398-399, I70.401-403, I70.408-409, I70.411-413, I70.418-419, I70.421-213, I70.428-429, I70.431-435, I70.438-439, I70.441-445, I70.448-45, I70.461-463, I70.468-469, I70.491-493, I70.498-499, I70.501-503, I70.508-509, I70.511-513, I70.518-519, I70.521-523, I70.528-529, I70.531-535, I70.538-539, I70.541-545, I70.548-549, I70.55, I70.561-563, I70.568-569, I70.591-593, I70.598-599, I70.601-603, I70.608-609, I70.611-613, I70.618-619, I70.621-623, I70.628-629, I70.631-635, I70.638-639, I70.641-645, I70.648-649, I70.65, I70.661-663, I70.668-669, I70.691-693, I70.698-699, I70.701-703, I70.708-709, I70.711-713, I70.718-719, I70.721-723, I70.728-729, I70.731-735, I70.738-739, I70.741-745, I70.748-749, I70.75, I70.761-763, I70.768-769, I70.791-793, I70.798-799, I70.8, I70.90-91, I74.01, I74.09-11, I74.19, I74.2-5, I74.8-9, I75.011-013, I75.019, I75.021-023, I75.029, I75.81, I75.89

© 2021 Carleton N et al. *JAMA Network Open*

Arterial injury	414.19, V12.50, 459.89, 414.10, 442.9, V12.59	I72.9, Z86.79, I25.3, I99.8
Deep vein thrombosis	415.11, 453.40, 453.42, 453.50, 453.52, 453.89, 997.2, 997.79, 998.83, 998.89, V12.51	I82.509, I82.5Z9, I82.409, T81.72XA, I82.401, I82.4Z9, I82.403, I82.90, T81.89XA, Z86.718
Pulmonary embolism	997.2, 997.79, 415.11, 415.19, 416.2, V12.55	I26.99, I27.82, T81.718A, Z86.711
Stroke	362.34, 430-432.1,432.9, 433.00-01, 433.10-11, 433.20-21, 433.30-31, 433.80-81, 433.90-91, 434.00-01, 434.10-11, 434.90-91, 435.0-3, 435.8-9, 438.0, 438.6-7, 438.9-10, 438.12-14, 438.19-22, 438.30-32, 438.40-42, 438.50-53, 438.81-85, 438.89, V12.54	H34.00-03, G45.0-3, G45.8-G46.2, I60.00-02, I60.10-12, I60.20-22, I60.30-32, I60.4-52, I60.6-I61.6, I61.8-I62.03, I62.1, I62.9-I63.00, I63.011-012, I63.019-020, I63.031-032, I63.039, I63.09-112, I63.119-12, I63.131-132, I63.139, I63.19-20, I63.211-212, I63.219, I63.22, I63.231-232, I63.239, I63.29, I63.30, I63.311-312, I63.319, I63.321-322, I63.329, I63.331-332, I63.339, I63.341-342, I63.349, I63.39-40, I63.411-412, I63.419, I63.421-422, I63.429, I63.431-432, I63.439, I63.441-442, I63.449, I63.49-50, I63.511-512, I63.521-522, I63.529, I63.531-532, I63.539, I63.541-542, I63.549, I63.59-6, I63.8-9, I65.01-03, I65.09, I65.21-23, I65.29, I65.8-9, I66.01-03, I66.09, I66.11-13, I66.19, I66.21-23, I66.29-3, I66.8-9, I67.841, I67.848, I69.00-023, I69.028, I69.031-034, I69.039, I69.041-044, I69.049, I69.051-054, I69.059, I69.061-065, I69.069, I69.090-093, I69.098, I69.10-11, I69.120-123, I69.131-134, I69.139, I69.141-144, I69.149, I69.151-154, I69.159, I69.161-165, I69.169, I69.190-193, I69.198, I69.20-223, I69.228, I69.231-234, I69.239, I69.241-244, I69.249, I69.251-252, I69.254, I69.259, I69.261-265, I69.269, I69.290-293, I69.298, I69.30-323, I69.328, I69.331-334, I69.339, I69.341-344, I69.349, I69.351-354, I69.359, I69.361-65, I69.369, I69.390-393, I69.398, I69.80-81, I69.821-823, I69.828, I69.831-834, I69.839, I69.841-844, I69.849, I69.851-854, I69.859, I69.861-865, I69.869, I69.891-893, I69.898, I69.90-91,

© 2021 Carleton N et al. *JAMA Network Open*

		I69.920-923, I69.928, I69.931-934, I69.939, I69.941-944, I69.949, I69.951-954, I69.959, I69.961-965, I69.969, I69.990-993, I69.998, Z86.73
Chronic obstructive pulmonary disease	427.31-32, 491.1, 491.20-491.22, 491.8-492.0, 492.8, 493.00-493.22, 494.0-1, 496	I480-I48.4, I48.91-2, J41.1, J41.8, J42, J43.0-3, J44.0-1, J44.9, J45.20-22, J45.20-22, J45.30-32, J45.40-42, J45.50-52, J47.0-1, J47.9
Fibromyalgia	729.1	M79.7
Psoriatic arthritis	696.3, 696.4, 696.5, 696.0, 696.1, 696.2, 696.8	L42, L30.5, L40.59, L40.0, L40.8, L94.5, L40.51, L40.53, L40.3, L40.4, L41.1, L41.5, L40.1, L40.50, L40.2, L41.3, L44.0, L40.52, L40.9, L41.0, L41.8, L44.8, L45, L41.4, L41.9, L40.54
Rheumatoid arthritis	714.0, 714.1, 714.81, 357.1, 359.6, 714.2, 714.89, 714.9	M05.542, M05.549, M05.572, M05.579, M05.412, M05.429, M05.459, M05.462, M05.711, M05.712, M05.721, M05.751, M05.769, M05.819, M05.842, M05.851, M05.862, M05.872, M06.071, M06.211, M06.239, M06.242, M06.252, M06.30, M06.332, M06.342, M06.351, M06.811, M05.00, M05.021, M05.039, M05.051, M05.20, M05.229, M05.251, M05.261, M05.262, M05.279, M05.30, M05.332, M05.60, M05.661, M05.671, M05.10, M05.142, M05.522, M05.552, M05.571, M05.441, M05.461, M05.472, M05.479, M05.731, M05.752, M05.779, M05.821, M06.00, M06.019, M06.021, M06.031, M06.059, M06.212, M06.219, M06.241, M06.261, M06.269, M06.271, M06.29, M06.312, M06.319, M06.349, M06.379, M06.842, M06.849, M06.9, M05.062, M05.079, M05.222, M05.249, M05.342, M05.351, M05.622, M05.632, M05.649, M05.679, M05.119, M05.169, M05.50, M05.532, M05.562, M05.59, M05.411, M05.431, M05.442, M05.49, M05.762, M05.829, M05.832, M05.859, M06.032, M06.039, M06.052, M06.062, M06.221, M06.231, M06.341, M06.361, M06.812, M06.821,

© 2021 Carleton N et al. *JAMA Network Open*

		M06.822, M06.872, M05.049, M05.052, M05.059, M05.071, M05.09, M05.211, M05.252, M05.349, M05.359, M05.369, M05.372, M05.611, M05.631, M05.651, M05.652, M05.122, M05.161, M05.19, M05.569, M05.419, M05.421, M05.729, M05.741, M05.761, M05.772, M05.811, M05.839, M05.852, M05.871, M06.049, M06.08, M06.249, M06.272, M06.369, M06.371, M06.829, M06.859, M06.871, M06.879, M05.012, M05.022, M05.221, M05.239, M05.242, M05.269, M05.272, M05.321, M05.621, M05.639, M05.519, M05.521, M05.529, M05.539, M05.739, M05.841, M05.89, M05.9, M06.011, M06.041, M06.042, M06.069, M06.09, M06.222, M06.229, M06.279, M06.311, M06.339, M06.38, M06.39, M06.831, M05.259, M05.311, M05.312, M05.319, M05.331, M05.361, M05.362, M05.629, M05.642, M05.659, M05.662, M05.121, M05.132, M05.139, M05.162, M05.171, M05.172, M06.4, M05.511, M05.531, M05.559, M05.432, M05.439, M05.451, M05.452, M05.719, M05.742, M05.749, M05.759, M05.79, M06.012, M06.022, M06.20, M06.251, M06.259, M06.321, M06.322, M06.329, M06.359, M06.362, M06.80, M06.852, M05.029, M05.041, M05.061, M05.212, M05.231, M05.241, M05.29, M05.322, M05.329, M05.112, M05.152, M05.541, M05.551, M05.561, M05.449, M05.471, M05.70, M05.771, M05.80, M05.849, M06.051, M06.061, M06.072, M06.079, M06.232, M06.28, M06.331, M06.819, M06.832, M06.841, M06.851, M06.861, M06.89, M05.019, M05.032, M05.042, M05.072, M05.219, M05.232, M05.271, M05.352, M05.379, M05.39, M05.619, M05.669, M05.672, M06.1, M05.111, M05.131, M05.141, M05.159, M05.512,

© 2021 Carleton N et al. *JAMA Network Open*

		M05.40, M05.422, M05.469, M05.722, M05.732, M05.812, M05.822, M05.831, M05.861, M05.869, M05.879, M06.029, M06.262, M06.352, M06.372, M06.839, M06.862, M06.869, M06.88, M05.011, M05.031, M05.069, M05.339, M05.341, M05.371, M05.612, M05.641, M05.69, M05.129, M05.149, M05.151, M05.179
Systemic lupus erythematosus	517.8, 289.81, 583.81, 424.91, 286.53, 420.0, 695.4, 710.0	M32.14, M32.9, D68.61, L93.1, M32.13, M32.15, M32.12, M32.11, L93.2, D68.62, L93.0, D68.312, M32.0, M32.10, M32.8, M32.19
Diabetes (includes both type 1 diabetes mellitus and type 2 diabetes mellitus; does not include diabetes insipidus)	T1DM: 250.01, 250.03, 250.41, 250.43, 250.51, 250.53, 250.61, 250.63, 250.71, 250.73, 250.81, 250.83<br>T2DM: 250.00, 250.02, 250.10-13, 250.20-23, 250.30-33, 250.40, 250.42, 250.50, 250.52, 250.60, 250.62, 250.70, 250.72, 250.80, 250.82, 250.90-93, V45.85, Z96.41	T1DM: E10.9, E10.21-22, E10.29, E10.36, E10.39-44, E10.49, E10.51-52, E10.59, E10.65, E10.69, E10.311, E10.319, E10.321, E10.329, E10.331, E10.339, E10.341, E10.349, E10.351, E10.359, E10.610, E10.618, E10.620-22, E10.628, E10.630, E10.638, E10.649<br>T2DM: E10.10-11, E10.641, E10.65, E10.69, E10.8, E11.00-01, E11.21-22, E11.29, E11.311, E11.319, E11.321, E11.329, E11.331, E11.339, E11.341, E11.349, E11.351, E11.359, E11.36, E11.39-44, E11.49, E11.51-52, E11.59, E11.610, E11.618, E11.620-22, E11.628, E11.630, E11.638, E11.641, E11.649, E11.65, E11.69, E11.8-9, E13.00-01, E13.10-11, E13.21-22 E13.29, E13.311, E13.319, E13.321, E13.329, E13.331, E13.339, E13.341, E13.349, E13.351, E13.359, E13.36, E13.39-44, E13.49, E13.51-52, E13.59, E13.610, E13.618, E13.620-22, E13.628, E13.630, E13.638, E13.641, E13.649, E13.65, E13.69, E13.8-9
Chronic kidney disease	585.3, 585.4, 585.5	N18.3, N18.4, N18.5

© 2021 Carleton N et al. *JAMA Network Open*

eTable 2. Calculation of Modified Charlson Comorbidity Index

The widely used Charlson comorbidity index (CCI) was adapted to calculate a modified Charlson comorbidity index (mCCI) to capture the extent to which the patients in this study had pre-existing conditions at the time of breast cancer diagnosis. Of note, in the original CCI, any solid tumor would give a patient a score of 2; because all patients in this study had a solid tumor (breast cancer), each patient's baseline score would be 2. The final mCCI score is a sum of the scores for each comorbid condition a patient had.

Charlson Comorbidity Index (CCI)	Modified Charlson Comorbidity Index (mCCI)	Score
Myocardial Infarction	Not captured.	1
Congestive Heart Failure	Included as is.	1
Peripheral Vascular Disease	Included using the following listings: vascular disease, arterial injury, DVT, or PE.	1
Cerebrovascular Disease	Included using the following listings: stroke.	1
Dementia	Not captured.	1
Chronic Obstructive Pulmonary Disease	Included as is.	1
Connective Tissue Disease	Included using the following listings: fibromyalgia, psoriatic arthritis, rheumatoid arthritis, and lupus.	1
Peptic Ulcer Disease	Not captured.	1
Mild Liver Disease	Not captured.	1
Diabetes	Included as is.	1
Any solid tumor	All patients had a solid tumor; thus all patients had a baseline score of 2.	2
Diabetes with Chronic Complications	Not captured.	2
Hemiplegia or Paraplegia	Not captured.	2
Renal Disease	Included using the following listings: chronic kidney disease.	2
Leukemia or lymphoma	Not captured.	2
Moderate or Severe Liver Disease	Not captured.	3
AIDS	Not captured.	6
Metastatic solid tumor	Not captured.	6

© 2021 Carleton N et al. *JAMA Network Open*

eTable 3. Baseline Characteristics of Patients

Patient characteristic		Total (N = 2,109)	No SLNB (n = 736)	SLNB (n = 1,373)	*P* value	No RT [a] (n = 848)	RT [a] (n = 1,219)	*P* value
Median age at diagnosis, years (Q1-Q3)		77.0 (73.0-82.0)	80.0 (74.0-86.0)	75.0 (72.0-79.0)	< 0.0001	80.0 (74.0-85.0)	75.0 (72.0-79.0)	< 0.0001
Median follow up time, years (Q1-Q3)		4.1 (2.5-5.7)	3.4 (1.7-5.1)	4.3 (3.1-6.0)	< 0.0001	3.4 (1.8-5.0)	4.5 (3.1-6.0)	< 0.0001
Insurance status								
	Commercial / Private	451 (21.3)	130 (17.7)	321 (23.4)	0.0091	143 (16.9)	299 (24.5)	0.0004
	Medicaid	33 (1.6)	13 (1.8)	20 (1.5)		16 (1.9)	14 (1.1)	
	Medicare	1,526 (72.4)	561 (76.2)	965 (70.3)		645 (76.1)	854 (70.1)	
	Not Insured	3 (0.1)	0 (0.0)	3 (0.2)		1 (0.1)	2 (0.2)	
	Other / Unknown	96 (4.6)	32 (4.3)	64 (4.7)		43 (5.1)	50 (4.1)	
Median tumor size (mm) (Q1-Q3)		15.0 (9.0-22.0)	15.0 (9.8-27.0)	14.0 (9.0-20.0)	< 0.0001	15.0 (9.5-25.0)	14.0 (9.0-20.0)	< 0.0001
Clinical T Stage								
	In Situ / T0	43 (2.0)	28 (3.8)	15 (1.1)	< 0.0001	17 (2.0)	24 (2.0)	0.0002
	T1	1,411 (66.9)	369 (50.1)	1042 (75.9)		470 (55.4)	915 (75.1)	
	T2 or Above	474 (22.5)	168 (22.8)	306 (22.3)		208 (24.5)	258 (21.2)	
	Unknown	181 (8.6)	171 (23.2)	10 (0.7)		153 (18.0)	22 (1.8)	
Grade								
	I	503 (23.9)	155 (21.1)	348 (25.3)	0.31	190 (22.4)	306 (25.1)	0.13
	II	1,186 (56.2)	410 (55.7)	776 (56.5)		466 (55.0)	693 (56.8)	
	III	288 (13.7)	99 (13.5)	189 (13.8)		129 (15.2)	154 (12.6)	
	NA / Unknown	132 (6.3)	72 (9.8)	60 (4.4)		63 (7.4)	66 (5.4)	
Pathologic node positivity after SLNB		--	--	159 (11.5)	--	39 (24.5) [b]	116 (73.0) [b]	--
Tumor histology								
	DCIS / IDC	1,447 (68.6)	488 (66.3)	959 (69.8)	0.55	591 (69.7)	849 (69.6)	0.93
	LCIS / ILC	278 (13.2)	96 (13.0)	182 (13.3)		117 (13.8)	157 (12.9)	
	Mixed IDC / ILC	68 (3.2)	21 (2.9)	47 (3.4)		29 (3.4)	37 (3.0)	
	Mucinous adenocarcinoma	62 (2.9)	26 (3.5)	36 (2.6)		26 (3.1)	38 (3.1)	
	Other / Unknown	254 (12.0)	105 (14.3)	149 (10.9)		85 (10.0)	138 (11.3)	
Surgical Intervention								
	SLNB	1,373 (65.1)	--	--	--	391 (46.1)	959 (78.7)	< 0.0001
	Lumpectomy	1,367 (64.8)	356 (48.4)	1011 (73.6)	< 0.0001	304 (35.8)	1039 (85.2)	< 0.0001
	Mastectomy	583 (27.6)	223 (30.3)	360 (26.2)	0.046	405 (47.8)	165 (13.5)	< 0.0001
	No Lumpectomy or Mastectomy	156 (7.4)	154 (20.9)	2 (0.1)	< 0.0001	137 (16.2)	14 (1.1)	< 0.0001
Therapy								
	Hormone therapy	1,786 (84.7)	578 (78.5)	1208 (88.0)	< 0.0001	651 (76.8)	1110 (91.1)	< 0.0001

© 2021 Carleton N et al. *JAMA Network Open*

Radiation therapy	1,219 (57.8)	260 (35.3)	959 (69.8)	< 0.0001	--	--	--
Chemotherapy	108 (5.1)	35 (4.8)	73 (5.3)	0.61	30 (3.5)	76 (6.2)	0.006
Modified Charlson comorbidity index (mCCI) Score							
2	875 (41.5)	251 (34.1)	624 (45.4)		320 (37.7)	544 (44.6)	
3	313 (14.8)	102 (13.9)	211 (15.4)		129 (15.2)	180 (14.8)	
4	79 (3.7)	39 (5.3)	40 (2.9)	< 0.0001	31 (3.7)	44 (3.6)	0.03
≥ 5	72 (3.4)	37 (5.0)	35 (2.5)		38 (4.5)	33 (2.7)	
NA	770 (36.5)	307 (41.7)	463 (33.7)		330 (38.9)	418 (34.3)	
mCCI Score, mean (SD)	2.5 (0.9)	2.7 (1.1)	2.5 (0.8)	< 0.0001	2.6 (1.0)	2.5 (0.8)	0.0112
Comorbidity [c]							
Congestive heart failure	72 (5.3)	43 (10.0)	29 (3.2)	< 0.0001	45 (5.3)	27 (2.2)	0.0002
Diabetes	230 (17.2)	84 (19.6)	146 (16.0)	0.61	91 (10.7)	136 (11.2)	0.78
Coronary artery disease	138 (10.3)	63 (14.7)	75 (8.2)	0.007	61 (7.2)	75 (6.2)	0.37
Stroke	91 (6.8)	45 (10.5)	46 (5.1)	0.005	46 (5.4)	43 (3.5)	0.047
COPD	99 (7.4)	37 (8.6)	62 (6.8)	0.59	46 (5.4)	52 (4.3)	0.25
Chronic kidney disease	48 (3.6)	22 (5.1)	26 (2.9)	0.13	20 (2.4)	26 (2.1)	0.76
Recurrence							
No Recurrence	2,028 (96.2)	703 (95.5)	1,325 (96.5)	0.28	801 (94.5)	1,186 (97.3)	0.002
Locoregional	28 (1.3)	8 (1.1)	20 (1.5)	0.55	22 (2.6)	6 (0.5)	< 0.0001
Distant	53 (2.5)	25 (3.4)	28 (2.0)	0.08	25 (2.9)	27 (2.2)	0.32
Surgical Site SLNB [d]							
Academic	571 (27.1)	131 (17.8)	440 (32.0)	< 0.0001	--	--	
Non-Academic	1,538 (72.9)	605 (82.2)	933 (68.0)		--	--	

Data are displayed as number (percentage) unless otherwise specified.
a There were n = 42 patients with unknown radiation therapy status.
b Calculated as a percentage of the total node positive cases (e.g., 39 patients out of 159 patients that were node positive after SLNB did not go on to have RT).
c Calculated as a percentage of those with comorbidity data (n = 1,339).
d Data from SLNB use in non-academic centers is aggregated from 14 different community hospitals, while data for the academic surgical site is two academic hospitals.
Statistical tests used: Mann-Whitney test, Chi-square test, Fisher's exact test.

© 2021 Carleton N et al. *JAMA Network Open*

eFigure 1. Flowchart for the Derivation of the Propensity Score Matched Cohorts

Flowchart for how the propensity score matched cohorts were derived. Cohort A, which was matched over the variables seen in the figure, was used to evaluate the results of SLNB. Cohort B, which was matched over the same variables as Cohort A with the addition of receipt of SLNB and the pathologic node status, was used to evaluate the results of RT.

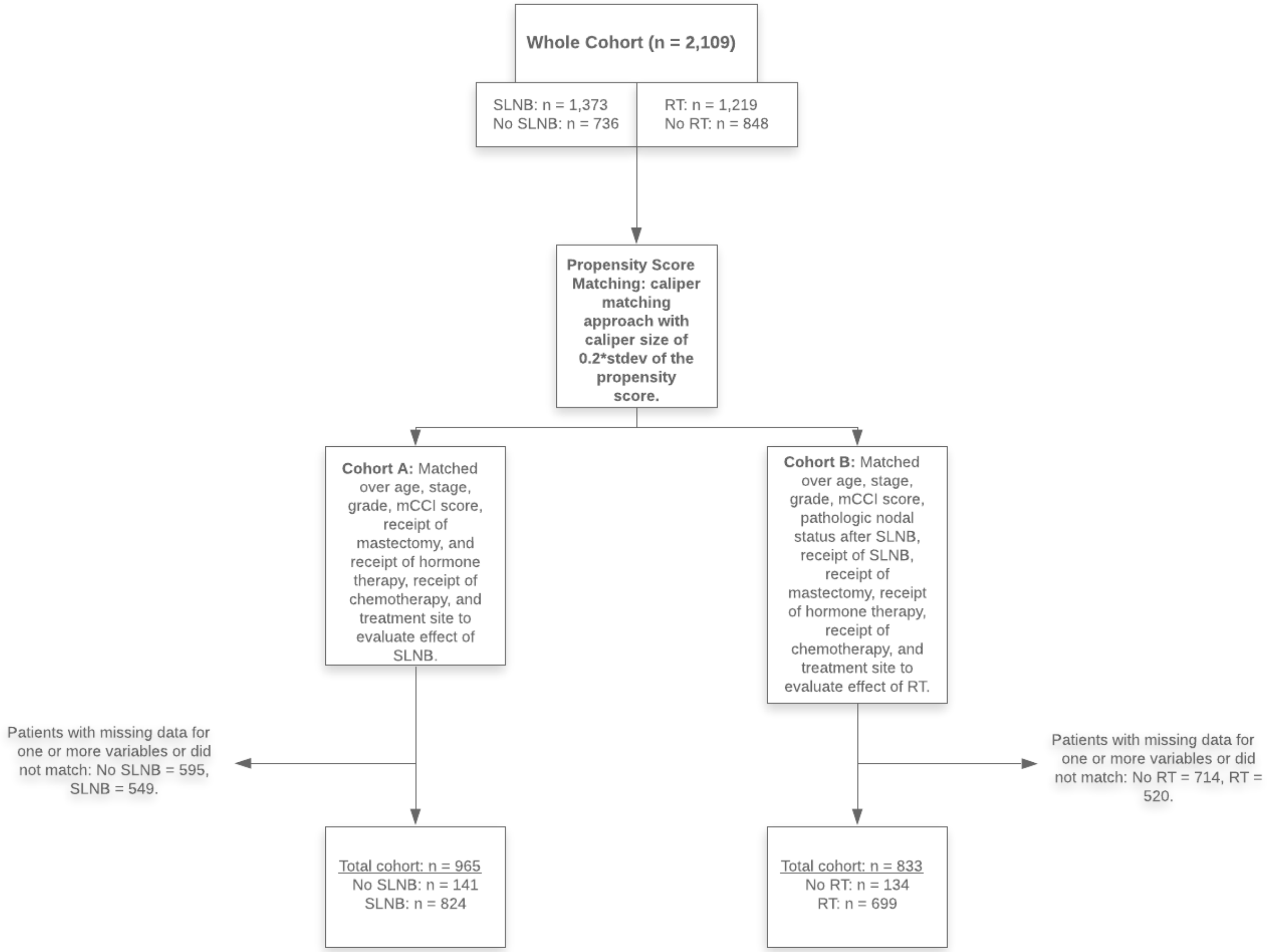

© 2021 Carleton N et al. *JAMA Network Open*

eFigure 2. Re-analysis of Cohort B Excluding Those Patients That Underwent Mastectomy

Using propensity score matched Cohort B, we now exclude patients who underwent mastectomy from the analysis. From the No RT group, n = 45 (36%) were excluded from the propensity matched cohort. However, since the No RT group was matched 1:5 with the RT group, this did not result in a significant loss in patients. From the RT group, n = 78 (11%) patients were excluded from the propensity matched cohort. We found that the data was very similar to that shown in Figure 3 and Table 2: RT does not impact DFS (p = 0.30 on log-rank testing) **(A)** or LRFS (p = 0.25 on log-rank testing) **(B)**; further, in multivariate analysis **(C)**, we found that RT did not significantly impact LRFS (p = 0.26) or DFS (p = 0.67). Further, after omitting mastectomy patients, we again find small absolute rates of recurrence (No RT group: 3.5% vs. RT group: 2.3%), supporting the fact that RT can be omitted about breast conserving surgery.

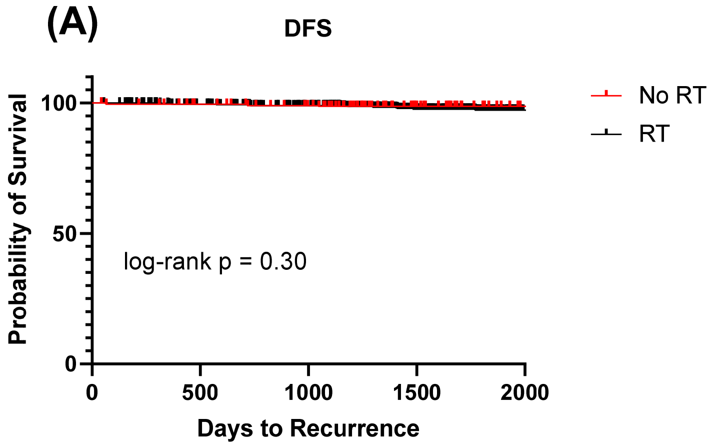

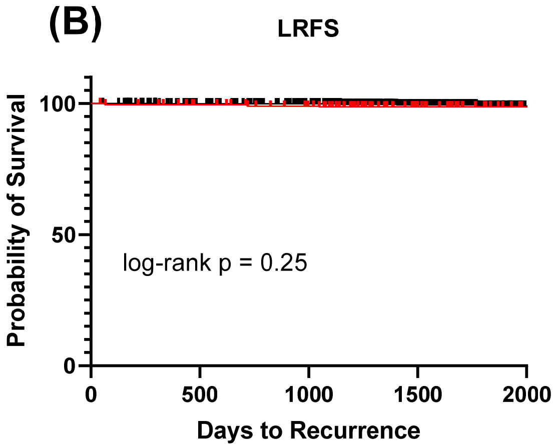

(C)

	LRFS		DFS	
Variable	HR (95% CI)	P-Value	HR (95% CI)	P-Value
Radiation Therapy	0.48 (0.14, 1.68)	0.26	1.23 (0.48, 3.18)	0.67
Pathologic Node Status*	--	--	1.73 (0.51, 5.93)	0.38
Age	1.21 (1.08, 1.35)	0.001	1.16 (1.07, 1.26)	< 0.0001
mCCI Score	0.82 (0.31, 2.17)	0.69	1.11 (0.66, 1.87)	0.70
Grade 2 & 3 vs. 1 Disease	0.64 (0.16, 2.62)	0.54	0.77 (0.25, 2.33)	0.64
T2 & Above vs. T1 Tumor	2.57 (0.64, 10.37)	0.18	3.34 (1.21, 9.25)	0.02

© 2021 Carleton N et al. *JAMA Network Open*
